# Resistance to the SDHI Fungicides Boscalid and Fluopyram in *Podosphaera xanthii* Populations from Commercial Cucurbit Fields in Spain

**DOI:** 10.3390/jof7090733

**Published:** 2021-09-08

**Authors:** Alejandra Vielba-Fernández, Álvaro Polonio, Laura Ruiz-Jiménez, Antonio de Vicente, Alejandro Pérez-García, Dolores Fernández-Ortuño

**Affiliations:** 1 Departamento de Microbiología, Facultad de Ciencias, Universidad de Málaga, 29071 Málaga, Spain; avielba@uma.es (A.V.-F.); polonio@uma.es (Á.P.); laura110493@uma.es (L.R.-J.); adevicente@uma.es (A.d.V.); aperez@uma.es (A.P.-G.); 2 Instituto de Hortofruticultura Subtropical y Mediterránea “La Mayora”, Departamento de Microbiología, Campus de Teatinos, Universidad de Málaga—Consejo Superior de Investigaciones Científicas (IHSM-UMA-CSIC), 29071 Málaga, Spain

**Keywords:** SDHI resistance, boscalid, fluopyram, disease control, fitness cost, fungicide resistance, powdery mildew, resistance development, resistance management, LAMP

## Abstract

Powdery mildew is caused by *Podosphaera xanthii*, and is one of the most important diseases that attacks Spanish cucurbit crops. Fungicide application is the primary control tool; however, its effectiveness is hampered by the rapid development of resistance to these compounds. In this study, the EC_50_ values of 26 isolates were determined in response to the succinate dehydrogenase inhibitor (SDHI) fungicides boscalid and fluopyram. From these data, the discriminatory doses were deduced and used for SDHI resistance monitoring during the 2018 and 2019 growing seasons. Of the 298 isolates analysed, 37.9% showed resistance to boscalid and 44% to fluopyram. Although different phenotypes were observed in leaf disc assays, the resistant isolates showed the same phenotype in plant assays. Compared to sensitive isolates, two amino acid changes were found in the SdhC subunit, A86V and G151R, which are associated mostly with resistance patterns to fluopyram and boscalid, respectively. Furthermore, no significant differences were observed in terms of fitness cost between the selected sensitive and resistant isolates analysed here. Lastly, a loop-mediated isothermal amplification (LAMP) assay was developed to detect A86V and G151R mutations using conidia obtained directly from infected material. Our results show that growers could continue to use boscalid and fluopyram, but resistance management practices must be implemented.

## 1. Introduction

Cucurbits are very important crops in Spanish agriculture. Within the vegetable sector, annual cucurbit production reached 3 million tons and yielded revenues of more than €1.9 billion in 2018 [1]. One of the most destructive diseases that affect these crops is powdery mildew, which is an important limiting factor for cucurbit production in Spain [2,3]. This disease can be caused by the biotrophic fungal species *Podosphaera xanthii* (Fr.) U Braun & N Shishkoff or *Golovinomyces cichoracearum* (DC.) VP Galut, [4], but in Spain, only *P. xanthii* has been detected over the last three decades [5,6,7,8,9,10]. Despite the substantial efforts that have been invested in plant breeding programs to combat powdery mildew disease, chemical control continues to be the principal practice for managing most cucurbit crops; however, it has been hampered by the emergence of resistant populations in the field soon after the introduction of certain classes of site-specific fungicides. In southern Spain, resistance to the most popular anti-powdery mildew fungicides, such as quinone outside inhibitors (QoIs), demethylation inhibitors (DMIs) and methyl benzimidazole carbamate (MBC) fungicides, has been reported [7,8,9,10,11]. More importantly, multiresistant isolates have been found in several areas of more intense cropping [9].

Succinate dehydrogenase inhibitors (SDHIs; FRAC group 7) have been on the market for more than 40 years, and are within the class with the fastest growth in terms of new compounds released onto the market. To date, twenty-three SDHI active ingredients belonging to 11 chemical classes with a broader spectrum of fungal activity have been offered for fungal plant pathogen control [12]. SDHI fungicides have a single-site mode of action, inhibiting the fungal respiration pathway by binding the ubiquinone binding site of succinate dehydrogenase (SDH; also known as complex II) and blocking mitochondrial electron transfer from succinate to ubiquinone [13]. This target protein is formed by four subunits (A, B, C and D), but the ubiquinone-binding site only comprises amino acids from subunits B (SdhB), C (SdhC), and D (SdhD; [14]).

SDHIs are classified as medium to high risk for resistance development. Therefore, it is not surprising that resistance to these fungicides has been documented since shortly after their registration for use against several phytopathogenic fungi [15]. More than 40 point mutations in SdhB, SdhC and SdhD have been linked to reduced sensitivity to SDHIs. In SdhB, the changes H272L/R/T and H277L/R/Y are the most common, having been described in several fungal plant pathogens, such as *Alternaria alternata* [16,17,18,19,20,21], *Botrytis cinerea* [22,23,24,25,26,27], *B. elliptica* [28], *Didymella bryionidae* [29], *P. xanthii* [30], *Pyrenophora teres* [31] and *Sclerotinia sclerotiorum* [28]. Notably, the amino acid changes H133R in SdhC and D124E/N and H133P/R in SdhD have been found in pathogens such as *A. alternata* [16,17,18,19,20,21], *A. solani* [32], *B. cinerea* [22], *P. xanthii* [33], *P. teres* [31] and *S. sclerotiorum* [28]. Although these amino acid changes are the most commonly described, other changes in the subunits SdhB (P225F/H/L/T, P230A/D/F/I/R, N230I, N235D/E/G/T and T268I), SdhC (S73P, N75S, G79R, T79N, W80S, A86V, N86S, G91R, S135R, H146R, G150R, H151R, V166M and G172D) and SdhD (A47T, S89P, G109V, S121P, H137R and D145G) have been documented in several phytopathogenic fungi, generating a pool of point mutations that confer different levels of resistance to the different SDHI fungicides [15]. 

To avoid field control failure, and for the efficient use of the fungicides that are available on the market, it is important to have good knowledge about the resistance situation in the field. For that reason, the most commonly used methods are based on mycelial growth or conidial germination in vitro assays in culture medium or plant material supplemented with different fungicide concentrations [34]. However, these methods are time consuming, especially when studying biotrophic fungi, and, therefore, molecular methods based on the detection of single-nucleotide polymorphisms are gaining in importance due to their quicker response times. Among the most commonly used approaches are polymerase chain reaction-restriction fragment length polymorphism (PCR-RFLP), allele-specific PCR (AS-PCR), cleaved amplified polymorphic sequences (CAPS) and high-resolution melt (HRM) analysis [16,17,35,36,37,38,39]. Although all these methods are faster than in vitro assays, they require specific equipment that not all laboratories can afford. In recent years, the loop-mediated isothermal amplification (LAMP) technique developed by Notomi and collaborators [40], has become an excellent alternative due to its cost, speed, and accuracy in fungicide resistance monitoring studies [41,42,43,44]. This technique, which is based on the combination of the *Bst* polymerase and four primer pairs, which hybridize with six regions in the target DNA, can amplify the product of interest under isothermal conditions [40]. In addition, the amplification product can be visualized with the naked eye using DNA-intercalating reagents such as SYBR-Green I [45], metal-ion indicators such as hydroxy naphthol blue (HNB) [46] or calcein [47], and even pH-sensitive dyes [48]. Recently, the LAMP technique has been successfully used to detect two-point mutations involved in SDHI resistance, namely H272R in SdhB in *B. cinerea* [49] and the change N75S in the SdhC in *C. cassiicola* [50]. 

In Spain, there are seven chemical classes of fungicides (aryl-phenyl-ketones (FRAC group 50), DMI (FRAC group 3), hydroxy-(2-amino-) pyrimidines (FRAC group 8), MBC (FRAC group 1), phenyl-acetamide (FRAC group U 06), QoIs (FRAC group 11) and SDHIs) registered for cucurbit powdery mildew control, with SDHI fungicides being one of the most frequently applied classes. Boscalid was the first SDHI registered in 2008, followed by fluopyram (2016), penthiopyrad (2017), isopyrazam (2018) and fluxapyroxad (2019). To date, *P. xanthii* resistance to SDHIs has not been documented in Spain, but evidence has started to emerge that resistance is developing in commercial cucurbit fields. For this reason, in the current study, cucurbit samples affected by powdery mildew symptoms from the primary cucurbit production areas in south-eastern Spain were collected during the 2018 and 2019 growing seasons. The fungal pathogen was isolated and identified as *P. xanthii*, and its sensitivity to boscalid and fluopyram was characterized using an in vitro leaf-disc bioassay and in planta analysis. The molecular alterations in the target gene subunits (SdhB, SdhC and SdhD) and the possible associated fitness costs were also studied. In addition, a LAMP assay for the rapid and reliable detection of two-point mutations related to *P. xanthii* SDHI resistance in our country was developed.

## 2. Materials and Methods

### 2.1. Fungal Isolates

In total, 26 single-spore isolates of *P. xanthii* were examined to determine the discriminatory doses for the SDHI fungicides boscalid and fluopyram (Table 1). These isolates were collected from several locations in Spain (Almeria, Badajoz, Ciudad Real, Cordoba, Granada, Malaga, Murcia, and Valencia) during the years 1988–2016, as previously described [7]. For the maintenance of the *P. xanthii* isolates, conidia were placed on zucchini cotyledons (*Curcubita pepo* cv. Negro Belleza; Semillas Fitó, Barcelona, Spain) that had previously been disinfected by 10 min of immersion in 5% sodium hypochlorite and deposited in 5 cm diameter Petri dishes containing Bertrand agar medium (sucrose 40 g/L, benzimidazole 0.03 mL/L, and agar 10 g/L in distilled water; [51]). Then, the isolates were maintained at 25 °C under a 16 h photoperiod of LED light or stored at −80 °C using silica gel for long-term conservation [52].

For SDHI monitoring studies, 298 *P. xanthii* isolates were collected from 18 fields and greenhouses located in four cucurbit production areas (Almeria, Granada, Malaga, and Murcia) in southeast Spain during the 2018 and 2019 growing seasons. At least 10 leaves affected by powdery mildew symptoms were taken per location. For all samples, single-spore isolation was performed as previously described [7]. The single-spore isolates were identified as *P. xanthii* according to the characteristics of the conidia and maintained at −80 °C [5,52].

### 2.2. Fungicide Sensitivity Studies for the SDHI Fungicides Boscalid and Fluopyram

To determine the discriminatory doses for distinguishing SDHI-sensitive from SDHI-resistant isolates, a leaf-disc bioassay was conducted with some modifications [7]. In brief, 1 cm diameter leaf discs from 8 to 10-day-old zucchini cotyledons (*Curcubita pepo* cv. Negro Belleza; Semillas Fitó, Barcelona, Spain) were cut with a corkborer and incubated upside down for 1 h on sterile filter paper that had absorbed 3 mL of sterile distilled water (untreated control) and several concentrations of the two study SDHIs. Five concentrations of boscalid (Cantus, BASF S.L., Ludwigshafen, Germany) and fluopyram (Luna Privilege; Bayer CropScience S.L., Leclair, France) were used at 0.01, 0.1, 1, 10 and 100 mg/L, with the last concentration being the field dose recommended by both manufacturers. Then, the discs were allowed to dry on Bertrand medium and the *P. xanthii* isolates were inoculated onto their adaxial surface using an eyelash. After 10 days of incubation under the same conditions described above, powdery mildew growth was assessed according to a 0–3 scale with 0 indicating the absence of symptoms and 1, 2 and 3 indicating <25%, 25–50% and >50% of the disc surface covered with fungal mycelial growth, respectively. To calculate the disease severity (DS), the formula [(0a + 1b + 2c + 3d)/3 N] × 100 was used, where a, b, c, and d correspond to the number of discs of scale value 0, 1, 2 and 3, respectively, and N was the total number of leaf discs assessed (a + b + c + d). Minimal inhibitory concentrations (MICs) were deduced directly from the data being the lowest concentration that inhibits the growth of *P. xanthii* after its inoculation. On the other hand, the fungicide concentrations inhibiting 50% of the powdery mildew growth (EC_50_) were calculated with graphical representation of the log transformation of percentages of inhibition (100-DS) and regression against the logarithm of the fungicide concentration used here [7]. The assay was performed three times. 

### 2.3. In Vivo Fungicide Sensitivity Tests to Boscalid and Fluopyram in Greenhouse Experiments

For the in vivo fungicide sensitivity assay, a total of 240 melon plants (*Cucumis melo* cv. Rochet; Semillas Fitó) were raised in seedling trays at a constant temperature (25 °C). A total of 16 *P. xanthii* isolates, which represented the most frequently found phenotypes observed during the SDHI monitoring studies, were tested: sensitive (S) to boscalid and fluopyram (SF9, 81210 and 18130304A); low resistance (LR) to both fungicides (18020307D); LR to boscalid and moderate resistance (MR) to fluopyram (18020307C); LR to boscalid and resistant (R) to fluopyram (18020307F); MR to both fungicides (18030306D); MR to boscalid and R to fluopyram (18020305L); R to boscalid and MR to fluopyram (18030306Q); R to both fungicides (18130301D, 18020303Q and 18030306H); LR to boscalid and high resistance (HR) to fluopyram (18020307E and 18020307J) and, lastly, R to boscalid and HR to fluopyram (18030306B). The following treatments were performed on each isolate: (i) five untreated plants, (ii) five plants treated with the field label rate of 100 mg/L boscalid (Cantus) and (iii) five plants treated with the recommended field dose of 100 mg/L fluopyram (Luna Privilege). The different applications were performed 24 h before the *P. xanthii* inoculation was performed. One leaf per plant was then inoculated with each isolate at three equidistant points using a paintbrush. Fifteen days after the inoculation, the development of each *P. xanthii* isolate on the leaf surface was evaluated. The experiment performed three times.

### 2.4. Determination of Mutations in the SdhB, SdhC and SdhD Genes

Fragments of the *SdhB*, *SdhC* and *SdhD* genes were searched in the partial transcriptome of the *P. xanthii* haustorium and mapped against the *P. xanthii* genome with TBLASTN using Blast Plus 2.2.30 and the NCR database of the NCBI in BAST1 with an e-value of 1 × 10^−5^ [53]. Once the sequences were obtained, several pairs of primers were designed, for the first time in *P. xanthii*, to amplify the open reading frames (ORFs) of the Sdh subunits B (SdhB_Forward/SdhB_reverse), C (SdhC_Forward/SdhC_Reverse), and D (SdhD_Forward/SdhD_Reverse; Table 2). The DNA of nine *P. xanthii*-sensitive isolates and 66 isolates found to have different levels of resistance to boscalid and fluopyram was extracted using the MasterPureTM Yeast DNA Purification Kit (Lucigen, Middleton, WI, USA). All PCRs were performed with Phusion High-Fidelity DNA Polymerase (Thermo Fisher Scientific, Vilnius, Lithuania) using the following mix: 1× HF Buffer (Thermo Fisher Scientific), 0.2 mM dNTPs (Bioline, Almería, Spain), 0.2 µM primers (Sigma-Aldrich, Taufkirchen, Germany), 0.5 U Phusion High-Fidelity DNA Polymerase (Thermo Fisher Scientific), 1 µL of *P. xanthii* DNA (100 ng/µL) and sterile distilled water up to a final volume of 50 µL. Amplifications were performed in an MJ Mini Thermal Cycler machine (Bio-Rad, Hercules, CA, USA) with an initial denaturation of 98 °C for 30 s; 35 cycles with three steps (denaturation at 98 °C for 10 s, hybridization at 61 °C for the *SdhB* and *D* subunits or 65 °C for *SdhC* for 30 s; and an extension step at 70 °C for 20 s); and lastly, an elongation step at 72 °C for 10 min. The amplified fragments were visualized in a 1% agarose gel stained with RedSafe (iNtRON, Burlington, MA, USA) and purified with a GFX PCR DNA and Gel Band Purification Kit (VWR, Barcelona, Spain). All the amplicons were sequenced by StabVida (Lisbon, Portugal), and the sequences were aligned using DNASTAR 7 computer sequence analysis software.

### 2.5. Fitness Assays

The following fitness components were investigated in six *P. xanthii* isolates that represent the most frequently found SDHI phenotypic groups: S (81210 and 18130304A); LR to boscalid and fluopyram (18020307D); MR to boscalid and R to fluopyram (18030305L); R to boscalid and fluopyram (18020303Q); and LR to boscalid and HR to fluopyram (18020307J). All the experiments were performed three times. 

#### 2.5.1. Conidial Germination

Conidial germination was measured at two different temperatures (17 and 23 °C). The isolates were inoculated by slightly blowing over the adaxial surface of three cucurbit cotyledons in a laminar air flow chamber under sterile conditions. The cotyledons were then deposited in 9 cm Petri dishes containing Bertrand medium and incubated for 24, 48 and 72 h in growth chambers with a 16 h photoperiod and the two temperatures described above. After the incubation period, four leaf discs were cut with an 11-mm diameter cork borer from each cotyledon. The discs were discoloured in boiling 96° ethanol for 15 min. The discoloured leaf discs were stained by soaking for 5 min with 150 µL of 0.01% Fluorescent Brightener 28 (Sigma-Aldrich, Burlington, MA, USA). Then, they were examined using a Nikon AZ-100 Multizoom Diascopic Microscope (UV-2A filter, EX 330-380; DM 400; BA 420, Tokyo, Japan). To determine the conidial germination status, ungerminated conidia, germinated conidia with one germination tube, and germinated conidia with two or more germinative tubes were considered [54]. Over 300 conidia per isolate, temperature and time were counted. The data were analysed by two-way ANOVA following Fisher’s LSD test (α ≤ 0.05), with a confidence interval (CI) of 95% using the software GraphPad Prism 8 (GraphPad Software, San Diego, CA, USA). Each experiment was carried out three times per isolate and condition.

#### 2.5.2. Mycelial Growth

Mycelial growth was determined by two different approaches: 

*Measurement of powdery mildew colony area*. Zucchini cotyledons were inoculated with four single conidia of each *P. xanthii* isolate using an eyelash and incubated in growth chambers at 17 °C for 30 days and at 23 °C for 15 days under a 16 h photoperiod. After these incubation times, photographs of the cotyledons were taken and the growth area of the colonies was measured using the image analysis software ImageJ 1.52a (Wayne Rasband, Nacional Institutes of Health, Bethesda, MD, USA). The pixel growth/total pixel ratio of the cotyledon was analysed for each temperature. This experiment was repeated three times for each isolate and condition. The data were analysed by one-way ANOVA following Fisher’s LSD test (α ≤ 0.05) with a confidence interval (CI) of 95% using the software GraphPad Prism 8 (GraphPad Software, San Diego, CA, USA).

*Quantification of the fungal mass by qPCR*. The biomass of the *P. xanthii* isolates grown over the zucchini samples described above was finely ground under liquid nitrogen. The total DNA was obtained using a MasterPureTM Yeast DNA Purification Kit (Lucigen) and quantified with a Nanodrop (Nanodrop ND-1000, Thermo Scientific). For *P. xanthii* DNA quantification, the *β-tubulin* gene (*PfTUB2*: KC333362.1) was amplified with the primer pair TubRT6F (5′-CTGCACCTCGCGAAACTAAC-3′) and TubRT6R (5′-CTACTAAACGCAGCGCAGTC-3′) as previously described [55]. For zucchini DNA quantification, the *actin* gene (*actin-7*: XM_008462689.2) was amplified using the primers Acting-F (5′-GGCTGGATTTGCCGGTGATGATGC-3′) and Acting-R (5′-GGAAGGAGGAAATCAGTGTGAACC-3′) as previously described Martínez-Cruz [56]. To generate a standard curve, serial dilutions (10^−1^ to 10^−5^) of genomic DNA (0.1–100 ng), extracted from noninoculated and inoculated zucchini cotyledons infected with the *P. xanthii* isolate SF9 were used as templates. After that, quantitative real-time PCR was conducted using a CFX384 Touch Real-Time PCR Detection System (Bio-Rad, Hercules, CA, USA). Amplifications were set up in a 10 µL volume containing 0.4 μL of each primer (TubRT6F/TubRT6R or Acting-F/Acting-R), 3.2 μL of water, 5 μL of EvaGreen SSoFast™ Supermix (Bio-Rad, Hercules, CA, USA) and 1 µL of genomic DNA (approximately 25 ng). Real-time PCRs were performed using the following parameters: 98 °C for 2 min, 40 cycles at 98 °C for 30 s and 60 °C for 30 s. For the melting curve analysis, the temperature was increased by 0.5 °C for 5 s, from 65 °C to 95 °C. The threshold cycle (Ct) values were calculated with Bio-Rad CFX Manager software V1.1 to identify significant fluorescence signals rising above background during the early cycles of the exponential growth phase of the PCR amplification process. A standard curve was drawn by plotting the natural log of the threshold cycle (Ct) against the concentrations of the dilution series for genomic DNA. The *PfTUB2* and *actin-7* gene copy numbers were calculated using the equation proposed by Whelan [57] and expressed as ng of total DNA. Data were analysed by one-way ANOVA following Fisher’s LSD test (α ≤ 0.05), with a confidence interval (CI) of 95%. For each sample, three replicates were analysed.

### 2.6. LAMP Technique

#### 2.6.1. LAMP Primer Design

To distinguish between *P. xanthii* SDHI-sensitive and SDHI-resistant isolates, a LAMP assay was developed. Four different sets of primers were generated to amplify the SdhC subunit carrying the amino acid change A86V (Set1 and 2; Table 2) and the mutation coding for the substitution G151R (Set3 and 4; Table 2). PrimerExplorer V5 software (https://primerexplorer.jp/e/, accessed on 13 August 2021) was used to develop the LAMP primers B3, F3, FIP and BIP. The FIP primers were composed of the complementary sequence of F1 (F1c) and F2, when the BIP primers included B1 (B1c) and B2 sequences. The last position of the 3′ ends of the B2 and F2 primers was designed to match the mutated nucleotide and, in addition, an extra mismatch was also added at the penultimate position to increase the specificity (Table 2).

#### 2.6.2. Mixture and Optimization of LAMP Reaction

To select the optimal set of primers for each amino acid substitution, a first screening was performed using DNA from the SDHI-sensitive isolate SF9 and the two resistant isolates with the A86V change (*P. xanthii* isolate 19020304D) and the G151R amino acid replacement (isolate 18020307I). The initial LAMP reaction was performed in a 10 µL volume containing 6 µL of GspSSD Isothermal Mastermix (ISO-001) (OpticGene, Horsham, UK), 1 µL of the primer mix (1.6 µM each of FIP and BIP, 0.2 µM each of F3 and B3), 1 µL of genomic DNA (approximately 100 ng) and 2 µL of sterile distilled water. During the first time, the amplification conditions were those recommended by the manufacturer: at 65 °C for 30 min with a melting curve analysis step (annealing curve 98–80 °C ramping at 0.05 °C per second). All the reactions were performed in Genie II (OpticGene), and the results were directly visualized in the same amplification platform through two values: the time to generate the amplified products, which is the time (min:seconds) of fluorescence emission when the LAMP product passes through the detection threshold, and the melting temperature of the primers, which is an indicator of sample contamination. After the results were analysed, Set1 (for the detection of the A86V change) and Set4 (for the G151R substitution) were selected for further analysis. Then, and for the optimization of the LAMP conditions, a gradient of temperatures (60.5–69.5 °C) was tested. When the optimal temperature was selected for each set, and to decrease the amplification time, the concentrations of the FIP and BIP primers were increased to 2 µM and remained the same (0.2 µM) for F3 and B3. After all these tests, the optimal conditions used for further LAMP assays were for Set1 (A86V), 63.8 °C for 16 min, and for Set4 (G151R), 62.7 C for 24 min. All the assays were performed three times.

#### 2.6.3. Specificity of LAMP

To test the specificity of the LAMP assay, genomic DNA from five different fungal species was used: *B. cinerea*, *D. bryoniae*, *Erysiphe diffusa*, *Macrophomina phaseolina* and *P. aphanis*. In addition, tubes with genomic DNA from the *P. xanthii* SDHIs-sensitive isolate SF9, the resistant 19020304D (SdhC-A86V) and 18020307I (SdhC-G151R), and sterile distilled water instead of DNA, were also included. The assay was carried out three times under the previously described LAMP conditions.

#### 2.6.4. Repeatability of LAMP

The repeatability of Set1 was tested in 8 *P. xanthii* isolates (18020303T, 19020304D, 18020305L, 18030306D, 18030306M, 18020307K, 18020307L and 18020307R) previously characterized as carrying the amino acid change A86V. Regarding Set4, this test was conducted with only two *P. xanthii* isolates (18030306K and 18020307I) obtained during SDHI field resistance monitoring studies, which carried the amino acid change G151R. In addition, four SDHI-sensitive isolates (MR03, SF9, SF60 and 311271) were also included. Tubes containing sterile distilled water instead of DNA, were used as negative controls. The reactions were performed under optimal conditions in triplicate.

#### 2.6.5. Optimization of LAMP Assay Using Spores as Template

To simplify the process of DNA extraction using conventional methods to obtain approximately 100 ng of total DNA, it is necessary to collect 50 mg of fresh fungal biomass from approximately two zucchini cotyledons infected with powdery mildew [7]. The LAMP assay was performed using DNA obtained from approximately 3 × 10^6^ *P. xanthii* spores as a template. In this case, DNA extraction was conducted as previously described by Zhu [50]. In brief, 30 µL of 10× Tris-EDTA (TE) buffer was used to collect spores from a mycelium colony by pipetting from two equidistant points of the infected cotyledon. Then, the volume was harvested into a 1.5 mL tube, boiled in sterile distilled water for 2 min, and incubated for 2 min on ice, with a final centrifugation at 12,000 rpm for 1 min. The resulting supernatant was used as a template in LAMP reactions following the optimized LAMP conditions, but in this case, and because the DNA quantity and quality were probably low, the reaction time was increased to 30 min. For Set1, DNA extracted from spores [50] and conventional methods (MasterPureTM Yeast DNA Purification Kit, Lucigen) from the SDHI-resistant isolate (19020304D; SdhC-A86V present) was tested. For Set4 the same strategy was employed using DNA from the SdhC-G151R-resistant isolate (18020307I). In addition, tubes including sterile distilled water and DNA extracted (from spores and conventional methods) from the sensitive *P. xanthii* isolate (SF9) were also tested.

#### 2.6.6. Testing LAMP Assay in Field Samples

To test the reliability of the LAMP technique, cucumber-infected leaves with powdery mildew disease collected from a greenhouse in Almeria were studied for LAMP reactions. Ten randomly distributed leaves were collected. *P. xanthii* colonies were selected from three equidistant points, and their spores were taken by pipetting 30 µL of 10× Tris-EDTA (TE) buffer. The fluid was then deposited in a microcentrifugation tube and processed to extract genomic DNA as previously described and according to Zhu et al. [50]. Later, DNA was LAMP-amplified with Sets 1 and 4. To confirm the LAMP results, in vitro fungicide sensitivity tests to boscalid and fluopyram were also performed.

## 3. Results

### 3.1. Determining the Discriminatory Doses to Boscalid and Fluopyram

To determine the discriminatory concentrations for the two SDHI fungicides, 26 randomly chosen *P. xanthii* isolates were tested using a leaf-disc bioassay, and the MIC and EC_50_ values were determined (Table 1). Based on the sensitivity results, the isolates were divided into three different groups for the two study fungicides. For boscalid, the first group was formed by 10 isolates with an MIC value of 1 mg/L and a mean EC_50_ value of 0.38 mg/L (0.03–0.97 mg/L), the second had six isolates with an MIC < 10 mg/L and EC_50_ value of 1.54 mg/L (0.62–3.5 mg/L) and, lastly, a third one with 10 isolates having an MIC of 10 mg/L and an EC_50_ value of 3.60 mg/L (0.20–11.17 mg/L). For fluopyram, the first, second and third groups were composed of eight isolates (MIC = 0.1 mg/L and EC_50_ value of 0.22 mg/L (3 × 10^−4^–0.77 mg/L)), 13 isolates (MIC = 1 mg/L and EC_50_ value of 0.47 mg/L (1 × 10^−4^–0.87 mg/L)), and five isolates (MIC < 10 mg/L and EC_50_ value of 2.20 mg/L (1.01–4.42 mg/L)), respectively. Therefore, and according to the data, 10 mg/L was the concentration chosen to perform SDHI field monitoring studies, but in addition to the detection *of P. xanthii* isolates with different levels of SDHI resistance, concentrations of 25 mg/L and 50 mg/L, and the recommended field dose of 100 mg/L, were also included for both fungicides [30].

### 3.2. SDHI Resistance Field Monitoring Studies

Two hundred and ninety-eight *P. xanthii* isolates were analysed for sensitivity to boscalid and fluopyram. Samples were taken from the provinces of Almeria (eight locations: D, E, F, and G in 2018 and J, K, L, and M in 2019), Granada (three locations: A, B and C in 2018), Malaga (Q and R in 2019) and Murcia (five locations: H and I in 2018 and N, O and P in 2019), representing some of the primary cucurbit production areas in Spain. The four discriminatory concentrations mentioned previously were tested. Based on their ability to grow on discs treated with these discriminatory doses, the *P. xanthii* isolates were grouped into different categories: (i) sensitive (S), showing no growth at any test concentration, (ii) low resistance (LR), with isolates able to grow at ≤25 mg/L (complete inhibition at 50 and 100 mg/L), (iii) moderate resistance (MR), with isolates able to develop until 50 mg/L (complete inhibition at 100 mg/L), (iv) resistant (R), able to grow at all tested concentrations but the mycelium did not completely cover the leaf disc and, finally, (v) high resistance (HR), with isolates able to grow vigorously at all analysed concentrations. 

**Figure 1:**
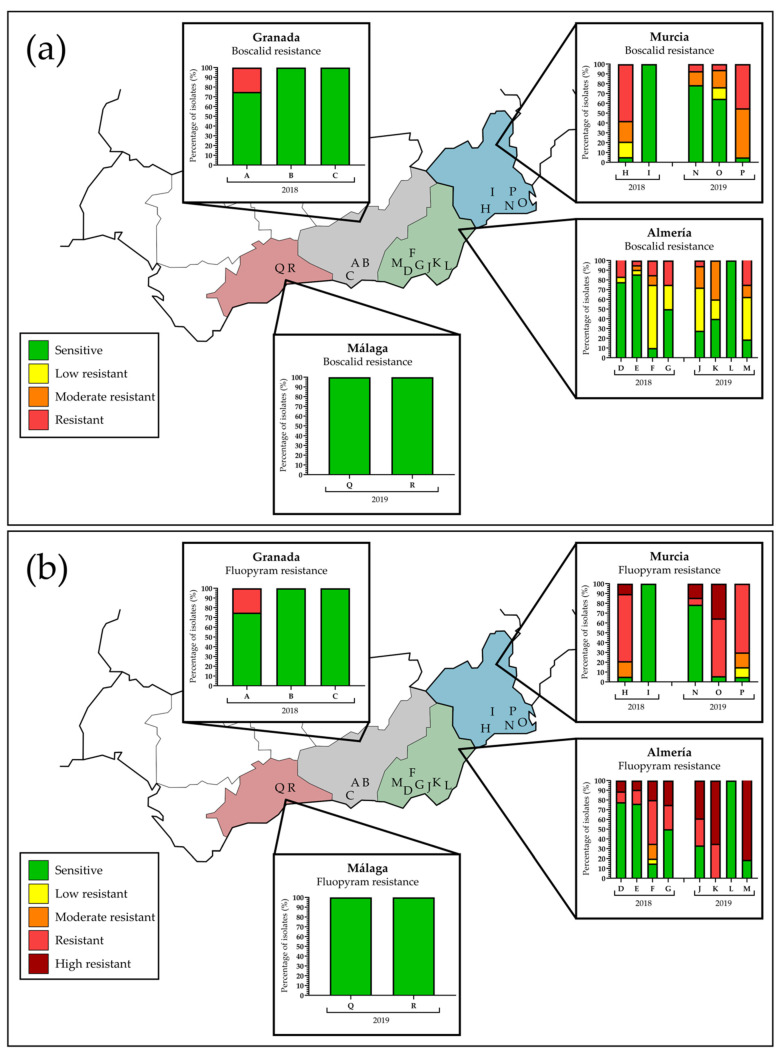
Percentage and phenotype of SDHI isolates detected in the SDHI field monitoring studies: (**a**) percentage of boscalid-sensitive and boscalid-resistant isolates in provinces of Almeria (eight localizations), Granada (three localizations), Malaga (two localizations) and Murcia (five localizations) during 2018–2019 seasons; (**b**) percentage of fluopyram-sensitive and fluopyram-resistant isolates in the same provinces and years.

The frequencies by localization, field, year, and phenotype for boscalid and fluopyram are shown in Figure 1 and Table 3 and Table 4. For boscalid (Table 3), the S group was formed by 185 isolates (96 in 2018 and 89 in 2019; 62.1%), and 113 *P. xanthii* isolates showed different levels of resistance (40 LR, 13.4%; 36 MR, 12.1% and 37 R, 12.4%). No boscalid-HR isolate was observed. The provinces of Malaga, which had no resistant isolates in 2019, and Granada, with two R isolates in 2018, showed the lowest percentages of boscalid resistance (0 and 0.7%, respectively), while the provinces of Murcia (with an overall 1.7% LR, 6.4% MR, and 7.4% R) and Almeria (11.7% LR, 5.7% MR, and 4.4% R isolates) had the highest frequencies of resistance (Table 3; Figure 1). For fluopyram (Table 4), 167 isolates (95 in 2018 and 72 in 2019; 56%) were sensitive, while 131 had some level of resistance: three isolates were LR, 9 MR, 67 R and 52 HR, representing 1, 3, 22.5 and 17.4%, respectively, of the *P. xanthii* population analysed here. With similar sensitivity results as boscalid, Malaga and Granada had the lower frequencies of resistant isolates (0 and 0.7%) and Almeria, followed by Murcia, with the highest frequencies (24.5 and 18.8%, respectively) (Table 4; Figure 1). In general, boscalid and fluopyram sensitivity remained stable during the two cucurbit seasons, although it should be noted that the levels of boscalid-MR isolates and fluopyram-HR isolates increased from 4.9 to 18.7% and from 7.7 to 26.5% in 2018 and 2019, respectively.

### 3.3. Fungicide Sensitivity Plant Assay

An inoculation of selected *P. xanthii* isolates was conducted on fungicide-sprayed and nonsprayed melon leaves to validate the results obtained during the in vitro fungicide sensitivity assays for boscalid and fluopyram. As expected, typical powdery mildew symptoms were observed on the leaves sprayed with distilled sterile water 15 days after *P. xanthii* inoculation. Regarding the fungicide-treated plants, the field rates of Cantus (boscalid) and Luna Privilege (fluopyram)-controlled S isolates (SF9, 81210 and 18130304A) but not the *P. xanthii* isolates that showed some level of resistance (LR, MR, R or HR) to the SDHI fungicides in in vitro tests, with no difference in the development of the different resistant phenotypes (data not shown). 

### 3.4. Analysis of SdhB, SdhC and SdhD Genes in P. xanthii Isolates

Gene fragments containing the ORFs of the three SDH subunits were found in the *P. xanthii* genome. For the ShdB subunit, the ORF had a length of 886 bp, which included two exons (345 bp and 471 bp) and one intron (70 bp), having a shared identity of 99.91% (e-value of 0) compared to the *P. xanthii ShdB* gene (LC522530.1) [58] and 92.65% (e-value of 2^−171^) compared to the ShdB protein of *Blumeria graminis* f. sp. *hordei* (CCU74871.1). For the ShdC subunit, the ORF was 737 bp in length, with three exons (43, 87 and 446 bp), two introns (66 bp and 95 bp) and shared identities of 100% (e-value of 0) and 76.15% (e-value of 5^−66^) with the *SdhC* gene of *P. xanthii* (LC522548.1) and the ShdC protein of *B. graminis* f. sp. *hordei* (CCU79401.1), respectively. Lastly, for the SdhD subunit, an ORF of 695 bp, which had two exons (223 bp and 353 bp) and one intron measuring 119 bp, was obtained. The nucleotide sequence mapped with the *P. xanthii SdhD* sequence (LC522550.1) had 99.98% shared identity (e-value of 0) and 76.15% shared identity (e-value of 5^−66^) with the SdhD protein of *B. graminis* f.sp. *hordei* (CCU79401.1). Using this information, the complete ORFs of the genes SdhB, SdhC and SdhD were PCR-amplified from 75 *P. xanthii* isolates (nine sensitive and 66 with different levels of resistance to boscalid and fluopyram) using the corresponding primer pairs listed in Table 2. Once the sequences obtained for the different subunits and isolates were analysed, no differences were found in the SdhB and SdhD subunits; however, two-point mutations were detected in SdhC. In this subunit, fifty-three isolates that showed different levels of resistance (LR, MR, R or HR) to boscalid and fluopyram and 11 *P. xanthii* isolates that were S to boscalid and R or HR to fluopyram presented an amino acid change of alanine to valine at position 86 (A86V; Table 5). However, the substitution of glycine for arginine at position 151 (G151R) was present in two isolates collected from Almeria and Murcia that were R to boscalid but S or LR to fluopyram (Table 5). The accession numbers of the SdhC sequences for four representative *P. xanthii* isolates carrying A86V and G151R amino acid changes were submitted to the DDBJ/EMBL/GenBank database (accession numbers MZ285078–MZ285081).

### 3.5. Fitness Cost of SDHI Resistance

To determine if the SDHI-resistant isolates had fitness penalties, two biological parameters, conidial germination and mycelium growth, were investigated at two temperatures (17 and 23 °C) and different times for the nine *P. xanthii* isolates (three S and six representing different levels of SDHI resistance).

**Figure 2:**
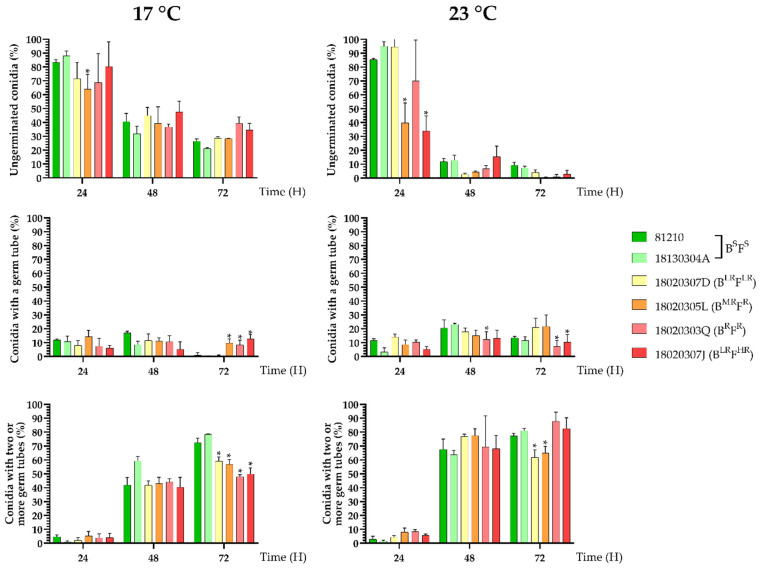
Percentage of ungerminated conidia, conidia with germ tube and conidia with two or more germ tubes incubated at 17 °C and 23 °C for 24, 48 and 72 h. *P. xanthii* isolates with different SDHI phenotypes (sensitive and low resistance, moderate resistance, resistant and high resistance to boscalid or fluopyram) were tested. For significantly different values, * is showed on the column.

For conidial germination, the number of ungerminated spores, germinated spores with a single germ tube, and spores with two or more germ tubes were observed for each isolate (Figure 2). In general, there were no differences between the SDHI-resistant isolates and the sensitive isolates, and the few significant differences did not follow any phenotypic pattern. For the ungerminated conidia, the number was higher at 24 h, and it was reduced at 48 h and 72 h, especially at 23 °C. The number of conidia with a germ tube remained below 20% three times, increasing slightly at 48 and 72 h at 23 °C. During this stage, and at 17 °C, three of the SDHI-resistant isolates (18020305L, 18020303Q and 18020307J) had significantly higher values, at approximately 10% cotyledon coverage, than the sensitive isolates (1% or less) at 72 h. Lastly, the percentage of conidia with two or more germ tubes, which are those that will normally develop haustoria and complete infection, increased notably at 48 and 72 h at both temperatures. At 17 °C, all the SDHI-resistant isolates had a percentage of spores with two or more germ tubes that was significantly lower than that of the sensitive isolates (78–72% for the sensitives versus 59–48% for the resistant ones), which was in contrast with the results of the spores with one germ tube at the same temperature after 72 h (Figure 2).

**Figure 3:**
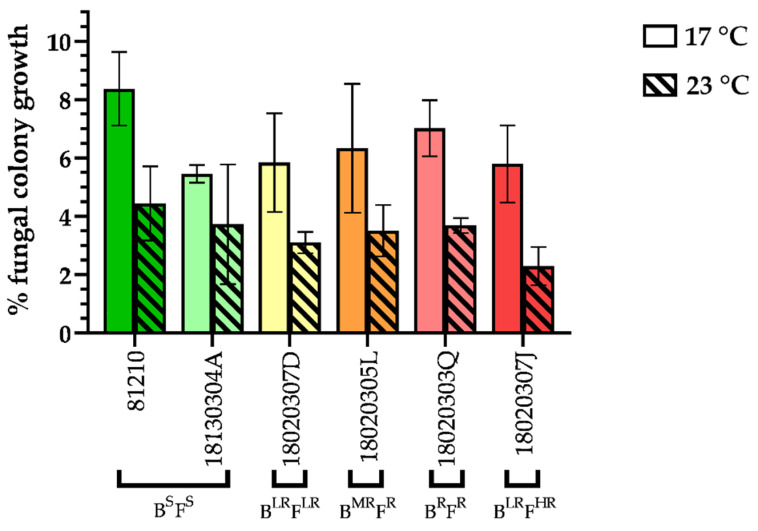
Percentage of fungal colony growth, starting from a single spore and covering the surface of a zucchini cotyledon. *P. xanthii* isolates with different SDHI phenotypes (sensitive (S) and low resistance (LR), moderate resistance (MR), resistant (R) and high resistance (HR) to boscalid (B) or fluopyram (F)) were incubated at 17 °C and 23 °C for 15 and 30 days, respectively.

The same trend accounted for mycelial growth, which was estimated by two different techniques, image analysis and qPCR. Using image analysis, the data shown in Figure 3 represent the mycelial growth rate in terms of leaf surface covered by powdery mildew and expressed as percentage of fungal colony growth. The comparison between SDHI-sensitive and SDHI-resistant *P. xanthii* isolates showed no significant differences between the two groups (α ≤ 0.05) (Figure 3). At 17 °C, the sensitive isolates showed a mean fungal colony growth of 8.4% (isolate 81210) and 5.5% for 18130304A, while the SDHI-resistant isolates had values of 5.8, 6.3, 7.0 and 5.8% for 18020307D (B^LR^F^LR^), 18020305L (B^MR^F^R^), 18020303Q (B^R^F^R^), and 18020307J (B^LR^F^HR^), respectively. The growth at 23 °C also remained similar between SDHI-sensitive and SDHI-resistant isolates. The mean values for 81210 and 18130304A were 4.4 and 3.7%, respectively, and the resistant isolates had similar results: 3.1% for 18020307D (B^LR^F^LR^), 3.5% for 18020305L (B^MR^F^R^), 3.7% for 18020303Q (B^R^F^R^) and 2.3% for 18020307J (B^LR^F^HR^). Regarding temperatures, the colonies covered more cotyledon surface at 17 °C than at 23 °C, which was explained by the different time of incubation of 30 days instead of 15 days. In addition, the quantification of the PfTUB2 gene copy number showed no significant differences (α ≤ 0.05) in the growth of SDHI-sensitive and SDHI-resistant (LR, MR, R and HR) isolates for the two temperatures tested here. Furthermore, no difference was observed in the amount of DNA in the same isolate between 17 and 23 °C. No pattern related to phenotype or temperature was observed, indicating that the amount of DNA in the colony does not depend on these two factors. For example, the DNA copy numbers obtained for the sensitive isolates 81210 and 18130304A and the resistant isolate 18020307J (B^LR^F^HR^) at 17 °C and 23 °C were below 1 × 10^10^, while 18020307D (B^LR^F^LR^) and 18020305L (B^MR^F^R^) had values above 2 × 10^10^ at both temperatures, even reaching 4 × 10^10^ DNA copies for 18020307D at 23 °C. The results obtained by image analysis and qPCR quantification were uniform.

### 3.6. LAMP Technique

#### 3.6.1. Primer Design and Optimization of Lamp Reactions

Four sets of primers, with two to detect *P. xanthii* SDHI-resistant isolates carrying the A86V amino acid change and two to detect the G151R substitution, were designed and tested. In the first case, both sets of primers (Set1 and Set2) were able to amplify the A86V-mutated isolate (19020304D) at 65 °C in 22 min, and thus we selected Set1 for further experiments. In the case of the G151R sets, only one of them (Set4) was able to generate positive results using DNA from the resistant isolate (18020307I) at 65 °C in 30 min. After that, the optimization of the LAMP reactions to detect both amino acid changes was performed. With this objective, three variables were checked: temperature, time, and primer concentrations. With respect to temperature and time, temperatures from 60.5 °C to 69.5 °C were tested over a 30-min period using the primer concentrations previously used in other studies (1.6 µM each of FIP and BIP, 0.2 µM each of F3 and B3; [11]). In relation to Set1, the temperature of 65 °C was selected because positive results were obtained in less time (22 min) using DNA from the A86V-resistant isolate (Table 6). Regarding Set4, a shorter time (24 min and 30 s) to obtain positive results was observed at 62.7 °C (Table 6). For both sets of primers, no results were obtained at 67.1 °C and 69.5 °C. The melting temperature (T) was similar (approximately 87 °C) for both sets at the different study temperatures, indicating the absence of contamination (Table 6). Once the optimal temperature was determined, and to decrease the amplification time, a higher concentration of the FIP and BIP primers was tested. For Set1, the concentrated primers were able to decrease the amplification time from 22 to 16 min; for Set4, it was reduced from 24 min and 30 s to 21 min and 30 s.

#### 3.6.2. Specificity of LAMP Using Different Fungal Species

Genomic DNA isolated from different fungal species (*B. cinerea*, *D. bryoniae*, *E. diffusa*, *M. phaseolina* and *P. aphanis*) was used to confirm the specificity of the LAMP assay. In addition, the sensitive *P. xanthii* isolate (SF9) and the SDHI-resistant isolates (19020304D and 18020307I) were also included. The LAMP reactions were performed using the optimized conditions described previously. No amplifications were obtained in the tubes containing sterile distilled water and the tubes with DNA extracted from the phytopathogenic fungi *B. cinerea*, *D. bryoniae*, *E. diffusa*, *M. phaseolina*, *P. aphanis* and the *P. xanthii* SDHI-sensitive isolate SF9. The LAMP reactions were positive (amplified product) in tubes containing genomic DNA from the 19020304D isolate (A86V present) at 65 °C in 15 min and 45 s for Set1 and the 18020307I isolate, carrying the G151R amino acid change, at 62.7 °C in 20 min and 30 s for Set4. The melting temperature was consistent with the positive controls.

#### 3.6.3. Repeatability of the LAMP Reaction

To confirm the reliability of the LAMP technique, a total of 14 *P. xanthii* isolates (four sensitive and 10 SDHIs-resistant), which were previously characterized in in vitro fungicide sensitivity assays and with a fragment of the sequenced SdhC subunit, were used. To characterize the A86V allele, seven isolates were included: 18020303T (B^R^F^R^), 18020305L (B^MR^F^R^), 18030306D (B^MR^F^MR^), 18030306M (B^MR^F^R^), 18020307K (B^LR^F^HR^), 18020307L (B^LR^F^R^), and 19020304D (B^LR^F^R^). To check the absence/presence of the G151R amino acid change, two *P. xanthii* isolates, obtained during the SDHI monitoring studies in 2018–2019, 18030306K (B^R^F^LR^) and 18020307I (B^R^F^S^), were studied. As expected, only the resistant isolates showed amplification with their corresponding set of primers (Table 6). All the isolates carrying the A86V mutation showed results between 14 and 16 min. The G151R isolates amplified in approximately 22 min. The T temperature was similar in all cases for both set of primers, at approximately 85 °C, indicating absence of the sample contamination (Table 6). Negative LAMP results (no amplification) were obtained using DNA extracted from the sensitive isolates (MR03, SF9, SF60 and 311271) and tubes without DNA as template (Table 6).

#### 3.6.4. Optimization of LAMP Assay Using Spores

Zucchini cotyledons infected with spores from SDHI-sensitive (SF9) and SDHI-resistant isolates carrying A86V (19020304D) and G151R (18020307I) amino acid changes were used. DNA was extracted with a quick assay using spores (approximately 3 × 10^6^) and with a conventional method. For Set1 (A86V), only the SDHI-resistant isolate 19020304D showed an amplification product using DNA extracted from spores and with conventional methods at 20 and 15 min, respectively. These results were consistent with those of Set4 (G151R), for which the amplification product was detected in the G151R-resistant isolate (18020307I) at 22 and 20 min using DNA extracted from spores or conventional methods, respectively. In both cases, the melting temperature was similar to the positive control, at approximately 85 °C (Table 6). For both sets, no SdhC amplifications were observed in tubes containing DNA from the SDHI-sensitive isolate (SF9) or when using sterile distilled water as a template.

#### 3.6.5. Testing LAMP Assay in Field Samples

Once the LAMP assay was optimized using a low number of spores, an assay was performed to confirm the reliability of the technique using plant material collected directly from the field. For that purpose, 10 powdery mildew-infected leaves were used to obtain DNA from spores and used for the LAMP reaction with the two primer sets. The results showed that *P. xanthii* isolates carrying only A86V were present in all samples tested, indicating resistance to SDHI fungicides. For Set1, the amplification products were obtained from 27 to 29 min in the different samples. The melting temperature was approximately 85 °C for all positive samples, indicating that the amplification product was not contaminated (Table 6). Notably, when fungal biomass obtained from the same leaves was tested using the in vitro leaf-disk sensitivity assay, positive results were obtained because the bulk conidial mass was able to grow at least at 50 mg/L of both boscalid and fluopyram, confirming the result obtained using the LAMP technique.

## 4. Discussion

The study of the fungicide resistance phenomenon is an essential step to avoiding the losses associated with fungal diseases in the field. The purpose of this work was to study, for the first time in Spain, the fungicide resistance situation of *P. xanthii* populations to SDHI fungicides, one of the families with relatively more active ingredients registered in recent years. For this purpose, several experiments, such as in vitro and in vivo fungicide sensitivity studies, analyses of the point mutations involved in resistance to boscalid and fluopyram, including its rapid molecular diagnosis through the LAMP technique and, lastly, the possible fitness cost associated with SDHI resistance, were performed.

The SDHI resistance monitoring studies performed during the 2018 and 2019 cucurbit production seasons showed that almost half of the isolates analysed had reduced sensitivity to boscalid (37.9%) and fluopyram (44.0%). The results varied in each test province: Granada and Malaga showed an absence or low levels of resistance, while Almeria and Murcia revealed high frequencies of resistant isolates (Almeria, 51.1% to boscalid and 57.4% to fluopyram; Murcia, 55.6% and 66.9% to boscalid and fluopyram, respectively). Moreover, the level of SDHI resistance in these two provinces usually increased from one year to another. In Almeria, the frequency of boscalid resistance increased from 42.9% to 59.4%, and for fluopyram it increased from 44.4% to 70.3%. With respect to Murcia, except for boscalid-resistant isolates, which remained at approximately 55%, the frequency of fluopyram resistance increased from 56.3% to 74.5%. According to the information provided by the growers, this increase could be due to the use of these fungicides to control other fungal diseases (anthracnose, Alternaria leaf blight, grey mould, leaf spot, and Sclerotinia stem rot). In addition, in the provinces of Granada and Malaga, which showed an absence or very low levels of resistance, a good fungicide management application, meaning the alternation between fungicides with different modes of action, and between single- and multisite fungicides, was performed.

Our results are supported by other studies in which high levels of SDHI resistance were described in several fungal pathogens, including *P. xanthii*, *B. cinerea*, *A. alternata*, *A. solari*, *D. bryoniae*, *C. cassiicola* and *P. teres* collected from fields where these fungicides have been frequently applied. In relation to *P. xanthii*, similar frequencies were described for boscalid in the American and Japanese populations of this pathogen, with 44 and 45.96% resistant isolates, respectively [30,58]. Different studies on *B. cinerea* showed approximately 50% boscalid-resistant isolates for the total population in Greece, Germany, and Spain [23,27,59]. With regard to a study developed in several strawberry fields in Spain, an increase from 5.3% to 10.4% in fluopyram-resistant isolates was observed in the *B. cinerea* population collected between 2015 and 2016 [27]. With reference to *A. alternata*, several studies in pistachio orchards in the USA also documented high percentages of SDHI-resistant isolates [19,60] and, in addition, an increasing trend over the years in farms where boscalid had been used extensively [16,61]. High frequencies of boscalid-resistant isolates have been described for *A. solani* (75%), D. bryoniae (79.6%), and *C. cassiicola* (48.9%) in monitoring studies performed in SDHI-treated fields from the USA and Japan [62,63,64]. Regarding *P. teres*, in a very complete study performed in several European countries, similar levels of resistance to those presented in this study, namely 44% for boscalid and 47% for fluopyram, were obtained for the isolates sampled from Germany in 2013 and 2014 [31]. Moreover, the overall percentage of SDHI-resistant isolates in all the studied countries increased from 1.2% to 25% in 2012 and 2013, respectively [31]. 

According to their growth in in vitro leaf-disc assays, our results showed that the Spanish *P. xanthii* population could be divided into four different levels of resistance; however, when some representative isolates were tested in planta, all the resistant phenotypes, regardless of the resistance category, were able to develop disease in plants sprayed with the field doses of boscalid and fluopyram (100 mg/L), showing the same colony development for all of them. The differences in SDHI applications may explain the different results observed in in vitro and in vivo assays. For *P. xanthii*, the laboratory approach for fungicide sensitivity tests is based on the use of leaf discs in direct contact with the fungicide solution during a period, which creates a larger exposure to the fungicide. This characteristic could make the final concentration of the fungicide in the plant tissue higher and make it possible to distinguish different categories of phenotypes. However, in the field, when these fungicides are applied, after entering the plant fungicides are transferred to different parts by the xylem due to acropetal phytomobility, making the concentration lower than that in leaf discs; therefore, all phenotypes (LR, MR, R and HR) were capable of developing powdery mildew disease under the conditions of these experiments [65]. Similar discrepancies have also been documented for another biotrophic fungus, the grape powdery mildew *Erysiphe necator* [66]. In that study, a leaf disc sporulation assay was conducted to establish sensitivity to quinoxyfen, with some isolates showing decreased sensitivity; however, when the results were contrasted with a quantitative assay based on germ tube elongation inhibition, the same isolates were completely inhibited by quinoxyfen [66]. Differences between resistant phenotypes in in vitro and in vivo assays were also observed in experiments on *B. cinerea*. Isolates that were considered moderately resistant and resistant to cyprodinil in the in vitro assay developed grey mould disease at the same levels in infected cyprodinil-treated fruits [67]. These results were confirmed in other studies in which *B. cinerea* isolates, which had moderate and higher resistance levels in in vitro assays for cyprodinil and iprodione, infected fruits with the same degree of virulence when treated with these fungicides [68,69]. 

Resistance to SDHI fungicides is conferred by point mutations in the three subunits, which conform to the ubiquinone binding site (SdhB, SdhC and SdhD) of mitochondrial complex II. Several amino acid changes have been described in different fungal species; [15]; however, little is known about powdery mildew fungi, with only three research studies, two on *E. necator* and one on *P. xanthii*. In *E. necator*, the amino acid change H242R/Y in ShdB correlated with boscalid and fluopyram resistance, and the amino acid change G169D in SdhD explained the low sensitivity to fluxapyroxad and fluopyram [70,71]. With respect to *P. xanthii*, a recent study documented some mutations in the subunits SdhD and SdhC. The amino acid change S121P in SdhD provided moderate levels of resistance to isopyrazam, penthiopyrad and pyraziflumid, while high levels of resistance to the same fungicides were conferred by the changes H137R, in the same subunit, and the changes G151R and G172D in SdhC. Lastly, high levels of resistance to isofetamid were associated with the presence of the amino acid change A86V in subunit SdhC [57]. In our study, this point mutation was observed in all *P. xanthii* isolates with resistance to boscalid and fluopyram but also in isolates that were only resistant to fluopyram, independent of the resistant phenotype observed in vitro. However, the amino acid substitution G151R was also observed in two *P. xanthii* isolates that presented resistance to boscalid and remained sensitive or low in resistance to fluopyram. Although boscalid and fluopyram were not tested in the work of Miyamoto and collaborators, other studies have explained the resistance to these two fungicides with the homologous position of these amino acid changes in other phytopathogenic fungi [72]. For example, in a study on the phytopathogenic fungus *Zymoseptoria tritici*, the substitution A84V was related to isofetamid and fluopyram resistance [73]. Furthermore, in a study by Scalliet and collaborators (2012), this change interacted with the fluopyram aliphatic linker, which is a characteristic of this compound [74]. In *B. cinerea*, A86V has been associated with resistance to fluopyram, but sensitivity to boscalid [75]. In C. cassiicola, the substitution S73P provided moderate levels of resistance to fluopyram [50]. In relation to the amino acid change G151R, the equivalence change G150R has been described in one *Sclerotinia homoeocarpa* isolate, which was resistant to several SDHI fungicides, such as boscalid, fluxapyroxad, isofetamid and penthiopyrad, but not to fluopyram [76]. 

Although the most frequent described mechanism of resistance is the presence of point mutations in the corresponding target genes, other mechanisms (detoxification, overexpression of the target genes or the implication of drug efflux transporters) could be involved. In our study, most of the *P. xanthii* isolates, which presented the amino acid change A86V, had cross-resistance to boscalid and fluopyram; however, eleven isolates with the same amino acid change were sensitive to boscalid and resistant to fluopyram. Therefore, could an alternative mechanism be involved in the SDHI resistance? The possibility was also raised in *Z. tritici* when several fluopyram- and isofetamid-resistant isolates did not carry any point mutations in the different Sdh subunits [73]. In other studies, *Z. tritici* isolates highly resistant to DMI fungicides and poorly resistant to QoIs and SDHIs presented overexpression in the BcMFS1 gene, which encodes a major facilitator transporter (MFS), a superfamily of transporters involved in a drug efflux system [77,78]. In the dollar spot fungus *S. homoeocarpa*, the overexpression of two ATP-binding cassette (ABC) drug efflux transporters (ShPDR1 and ShartD) explained the reduced sensitivity to nonrelated, site-specific fungicides, including boscalid (SDHI), iprodione (dicarboxamide) and propiconazole (DMI) [79]. In the same pathogen, the amino acid substitution M853T in the transcription factor ShDR1 was responsible for the overexpression of the ABC transporter, resulting in fungicide resistance to propiconazole (DMI fungicide), iprodione (dicarboximide) and boscalid (SDHI fungicide; [80]). In the wheat powdery mildew *B. graminis* f. sp. *tritici*, a BgABC1 gene was related to the overexpression of the ABC transporter in seeds treated with the DMI fungicide triadimefon [81]. In relation to *P. xanthii*, there is no information about the correlation of ABC or MFS transporter expression and fungicide resistance. However, the genome of this organism has recently been published [52] and the implication of some of these transporter superfamilies in resistance to different fungicides, including SDHIs, could be explored in future studies.

An important part of resistance analysis is the biological cost that may be associated with different processes involved in the natural survival of the pathogen. The characterization of this cost is essential to predicting the behaviour of the entire pathogen population and to implementing disease control strategies in the future [82]. This possible biological cost is usually studied, among other approaches, based on sporulation, mycelial growth or the aggressiveness of the study isolates [83,84]. The results observed in the present study showed no fitness cost in a collection of representative SDHI-sensitive and SDHI-resistant *P. xanthii* isolates on mycelial development and spore germination. In studies on other phytopathogenic fungi, such as *A. alternata*, similar results were obtained, and no differences were observed between germination, hyphal development, sporulation or virulence when boscalid-sensitive and resistant isolates were compared [61]. In addition, the boscalid resistance levels did not decrease after various subcultures in the absence of this fungicide, indicating the stability of resistance without selection pressure [61]. Similar results were observed in *A. solani*, with no significant differences in spore germination, mycelial expansion or aggressiveness in in vivo tests among sensitive and resistant isolates to several fungicides (anilinopyrimidine [AP], QoI and SDHI, [84]). 

Fungicide resistance monitoring studies on cucurbit powdery mildew are usually performed using bioassays with plants, meaning there is a great investment of time and material [34]. However, when the mechanism of resistance is known and is caused by point mutations in the corresponding target gene, the detection of the different phenotypes can be performed using molecular methods, making it a better solution than time-consuming bioassays. In recent years, the LAMP technique has become an interesting alternative that offers the possibility of obtaining results from field samples within a few hours, and is an attractive tool to use in resistance monitoring studies. In the present study, this technique was developed to detect the two-point mutations (A86V and G151R) observed in the SDHI-resistant population of *P. xanthii* in less than 40 min, complementing a previous study on the detection of MBC-resistant isolates carrying the E198A substitution in cucurbit powdery mildew [10]. All this information will help to provide faster responses to growers regarding the effectiveness of these fungicides in fields affected with this disease.

The registration of SDHI fungicides, resistance to this class of fungicides in *P. xanthii* has been described in several countries [85]. Moreover, high levels of resistance to other nonrelated fungicides have been reported previously in this pathogen in Spain and in other parts of the world [7,8,9,57,86,87,88,89]. In 2020, the European Committee approved the European Green Deal, which proposes the promotion of an efficient use of resources, the restoration of biodiversity, and the reduction of pollution, toward a climate-neutral Europe by 2050. This change would be achieved through a series of objectives, including reducing pesticide use by 50% [90]. This reduction is being reflected in Spain and, since 2017, five active substances belonging to three different chemical families [cyproconazole, flutriafol and hexaconazole (DMIs), kresoxim-methyl (QoIs) and quinoxyfen (aza-naphthalenes)] have been withdrawn from use. Due to the imminent reduction in chemical tools to control fungal diseases, the time needed to generate new substances from the phytosanitary sector (with a mean of ten years), and the rapid development of resistance by this and other fungal plant pathogens, it is fundamental to perform monitoring studies for testing the efficacy of each fungicide to increase the effectiveness of these compounds over time and to slow down the emergence of resistance. The implementation of integrated pest management (IPM) with, among others, the alternation between single-site and multisite fungicides, changes the mode of action between single-site fungicide families and is a good practice in the field. This may control the rise of resistance to SDHI and other families of fungicides, which is currently necessary.

## Figures and Tables

**Table 1:** Sensitivity of 26 randomly chosen *Podosphaera xanthii* isolates to the SDHI fungicides boscalid and fluopyram. The values of the minimal inhibitory concentration (MIC) and concentration inhibiting 50% of the growth (EC_50_) are shown.

Isolate	Year	Location	Host	MIC (mg/L)		EC_50_ (mg/L)	
				Boscalid	Fluopyram	Boscalid	Fluopyram
22014	2002	Almeria	Zucchini	1	0.1	0.36	0.13
31430	2003	Murcia	Melon	10	1	11.17	0.70
31869	2004	Murcia	Melon	<10	<10	0.62	1.01
44675	2003	Valencia	Watermelon	<10	0.1	1.78	0.04
64132	2002	Cordoba	Zucchini	<10	0.1	1.10	0.66
71175	2002	Ciudad Real	Melon	<10	<10	1.27	1.66
72174	2002	Ciudad Real	Zucchini	10	1	3.42	0.45
81210	2002	Badajoz	Melon	1	0.1	0.03	3 × 10^−4^
221104	2006	Almeria	Zucchini	<10	<10	1	1.91
311254	2008	Murcia	Melon	1	0.1	0.40	0.19
311271	2008	Murcia	Melon	10	0.1	2	0.77
711356	2008	Ciudad Real	Melon	10	1	1.51	0.24
711419	2009	Ciudad Real	Melon	1	1	0.26	0.60
711420	2009	Ciudad Real	Melon	<10	0.1	3.50	2 × 10^−3^
811414	2009	Badajoz	Melon	1	0.1	0.54	2 × 10^−3^
811415	2009	Badajoz	Melon	10	<10	5.46	4.42
1502404 A	2016	Almería	Watermelon	10	1	5.86	0.47
1503405 C	2016	Murcia	Watermelon	10	1	2.33	0.43
1509409 C	2015	Murcia	Watermelon	10	1	1.11	1 × 10^−4^
1513406 C	2015	Granada	Watermelon	10	1	2.94	0.52
JF01′12	2012	Ciudad Real	Melon	1	1	0.97	0.77
JF02′11	2011	Ciudad Real	Melon	1	1	0.42	0.87
JF06′10	2010	Ciudad Real	Melon	1	1	0.24	0.55
JF09′11	2011	Ciudad Real	Melon	1	1	0.52	0.35
JF13′10	2010	Ciudad Real	Melon	10	1	0.20	0.17
SF9	1988	Malaga	Zucchini	1	<10	0.08	2.01

**Table 2:** Primers used in this study.

Primer Name		Sequence (5′-3′)	Description
SdhB_Forward		GCGGGGAGACCTCTGAGATA	Used to amplify a fragment of 1060-bp which contains the 886-bp *sdhB* ORF of *P. xanthii*
SdhB_Reverse		GCCAGCAAGGGAGGATGATAA	
SdhC_Forward		CCAATTCTCGCCGATTTCGC	Used to amplify a fragment of 1220-bp which contains the 737-bp *sdhC* ORF of *P. xanthii*
SdhC_Reverse		CCCGCATACCCCTGGTATTC	
SdhD_Forward		CGGGTAGGTCGCCTTAGTAC	Used to amplify a fragment of 1079-bp which contains the 695-bp *sdhD* ORF of *P. xanthii*
SdhD_Reverse		CGACGTGTCGCATTTGCATT	
LAMP assay			
Set1	F3	ATCAACGTGACGACCTGA	Set of primers used in LAMP assays to amplify a fragment of 189-bp of the *SdhC* allele coding for the A86V amino acid change.
	B3	CCACCCGATATGACACAG	
	FIP	GGTTCTTACGTTGAGCTATAAGAGTCTCTTAGACCCGTGACAAC	
	BIP	GTCCCACATCTCCGCATTTACCCCGTAATGCGATTCAGgA ^1^	
Set2	F3	ATCAACGTGACGACCTGA	
	B3	CCACCCGATATGACACAG	
	FIP	GGTTCTTACGTTGAGCTATAAGAGTTTTTCTCTTAGACCCGTGACAAC	
	BIP	GTCCCACATCTCCGCATTTACTTTTCCCGTAATGCGATTCAGgA ^1^	
Set3	F3	CTAGGATTGAAGTCTCTGGT	Set of primers used in LAMP assays to amplify a fragment of the 199-bp of the *SdhC* allele coding for the G151R amino acid change.
	B3	TTTGTAGAGCCTACGTGATT	
	FIP	TTGAAAAGGCCTTGCCCAAATCCCTTTCACTTTTCATTCAATAAAaA ^1^	
	BIP	AGGCAGTTATTAAAACAGGCTGGGTAACCAAAGCTAATGCACT	
Set4	F3	CTAGGATTGAAGTCTCTGGT	
	B3	TTTGTAGAGCCTACGTGATT	
	FIP	TTGAAAAGGCCTTGCCCAAATCTTTTCCTTTCACTTTTCATTCAATAAAaA ^1^	
	BIP	AGGCAGTTATTAAAACAGGCTGGTTTTGTAACCAAAGCTAATGCACT	

^1^ The nucleotide that hybridizes the amino acid change is in underline and the additional mismatch in lowercase.

**Table 3:** Number and frequencies of boscalid-sensitive and -resistant *P. xanthii* isolates collected from four different cucurbit production areas in Spain during 2018 and 2019 growing seasons.

2018 (*N* = 143)							2019 (*N* = 155)						
Field	Location	Phenotype					Field	Location	Phenotype				
		S	LR	MR	R	HR			S	LR	MR	R	HR
D	Almeria	14 (77.8%)	1 (5.6%)	0	3 (16.7%)	0	J	Almeria	5 (27.8%)	8 (44.4%)	4 (22.2%)	1 (5.6%)	0
E		18 (85.7%)	1 (4.8%)	1 (4.8%)	1 (4.7%)	0	K		8 (40.0%)	4 (20.0%)	8 (40.0%)	0	0
F		2 (10.0%)	13 (65.0%)	2 (10.0%)	3 (15.0%)	0	L		10 (100%)	0	0	0	0
G		2 (50.0%)	1 (25.0%)	0	1 (25.0%)	0	M		3 (18.8%)	7 (43.8%)	2 (12.5%)	4 (25.0%)	0
H	Murcia	1 (5.3%)	3 (15.8%)	4 (21.1%)	11 (57.9%)	0	N	Murcia	11 (78.6%)	0	2 (14.3%)	1 (7.1%)	0
I		13 (100%)	0	0	0	0	O		11 (64.7%)	2 (11.8%)	3 (17.6%)	1 (5.9%)	0
							P		1 (5.0%)	0	10 (50.0%)	9 (45.0%)	0
A	Granada	6 (75.0%)	0	0	2 (25.0%)	0	Q	Malaga	20 (100%)	0	0	0	0
B		20 (100%)	0	0	0	0	R		20 (100%)	0	0	0	0
C		20 (100%)	0	0	0	0							
	Total	96 (67.1%)	19 (13.3%)	7 (4.9%)	21 (14.7%)	0		Total	89 (57.4%)	21 (13.5%)	29 (18.7%)	16 (10.3%)	0

Phenotypes are indicated as: S, sensitive; LR, low resistance; MR, moderate resistance; R, resistant; HR, high resistance.

**Table 4:** Number and frequencies of fluopyram-sensitive and -resistant *P. xanthii* isolates collected from four different cucurbit production areas in Spain during 2018 and 2019 growing seasons.

2018 (*N* = 143)							2019 (*N* = 155)						
Field	Location	Phenotype					Field	Location	Phenotype				
		S	LR	MR	R	HR			S	LR	MR	R	HR
D	Almeria	14 (77.8%)	0	0	2 (11.1%)	2 (11.1%)	J	Almeria	6 (33.3%)	0	0	5 (27.8%)	7 (38.9%)
E		16 (76.2%)	0	0	3 (14.3%)	2 (9.5%)	K		0	0	0	7 (35.0%)	13 (65.0%)
F		3 (15%)	1 (5.0%)	3 (15.0%)	9 (45.0%)	4 (20.0%)	L		10 (100%)	0	0	0	0
G		2 (50%)	0	0	1 (25.0%)	1 (25.0%)	M		3 (18.8%)	0	0	0	13 (81.3%)
H	Murcia	1 (5.3%)	0	3 (15.8%)	13 (68.4%)	2 (10.5%)	N	Murcia	11 (78.6%)	0	0	1 (7.1%)	2 (14.3%)
I		13 (100%)	0	0	0	0	O		1 (5.9%)	0	0	10 (58.8%)	6 (35.3%)
							P		1 (5.0%)	2 (10%)	3 (15.0%)	14 (70.0%)	0
A	Granada	6 (75%)	0	0	2 (25.0%)	0	Q	Malaga	20 (100%)	0	0	0	0
B		20 (100%)	0	0	0	0	R		20 (100%)	0	0	0	0
C		20 (100%)	0	0	0	0							
	Total	95 (66.4%)	1 (0.7%)	6 (4.2%)	30 (21.0%)	11 (7.7%)		Total	72 (46.5%)	2 (1.3%)	3 (1.9%)	37 (23.9%)	41 (26.5%)

Phenotypes are indicated as: S, sensitive; LR, low resistance; MR, moderate resistance; R, resistant; HR, highly resistant.

**Table 5:** Fluopyram and boscalid MIC values, phenotype, and amino acid substitution in the SdhC subunit in *P. xanthii* isolates collected during the 2018 and 2019 cucurbit growing seasons in Spain.

Isolate	Location	Host	MIC Value (mg/L)		Phenotype		Amino Acid Substitution
			Boscalid	Fluopyram	Boscalid	Fluopyram	
18130301D	Granada	Cucumber	>100 ^1^	>100 ^1^	R	R	A86V
18130301E	Granada	Cucumber	>100 ^1^	>100 ^1^	R	R	A86V
18020303M	Almeria	Cucumber	>100 ^1^	>100 ^2^	R	HR	A86V
18020303Q	Almeria	Cucumber	>100 ^1^	>100 ^1^	R	R	A86V
18020303S	Almeria	Cucumber	<50	>100 ^2^	LR	HR	A86V
18020303T	Almeria	Cucumber	>100 ^1^	>100 ^1^	R	R	A86V
18020305A	Almeria	Cucumber	0	>100 ^1^	S	R	A86V
18020305K	Almeria	Cucumber	<50	>100 ^2^	LR	HR	A86V
18020305L	Almeria	Cucumber	<100	>100 ^1^	MR	R	A86V
18020305P	Almeria	Cucumber	0	>100 ^2^	S	HR	A86V
18020305S	Almeria	Cucumber	>100 ^1^	>100 ^1^	R	R	A86V
18030306A	Murcia	Cucumber	<100	>100 ^1^	MR	R	A86V
18030306B	Murcia	Cucumber	>100 ^1^	>100 ^2^	R	HR	A86V
18030306C	Murcia	Cucumber	>100 ^1^	>100 ^1^	R	R	A86V
18030306D	Murcia	Cucumber	<100	<100	MR	MR	A86V
18030306E	Murcia	Cucumber	0	>100 ^1^	S	R	A86V
18030306F	Murcia	Cucumber	>100 ^1^	>100 ^1^	R	R	A86V
18030306G	Murcia	Cucumber	>100 ^1^	>100 ^1^	R	R	A86V
18030306H	Murcia	Cucumber	>100 ^1^	>100 ^1^	R	R	A86V
18030306I	Murcia	Cucumber	<100	<100	MR	MR	A86V
18030306J	Murcia	Cucumber	>100 ^1^	>100 ^1^	R	R	A86V
18030306K	Murcia	Cucumber	>100 ^1^	<50	R	LR	G151R
18030306L	Murcia	Cucumber	<50	>100 ^1^	LR	R	A86V
18030306M	Murcia	Cucumber	<100	>100 ^1^	MR	R	A86V
18030306N	Murcia	Cucumber	<50	>100 ^2^	LR	HR	A86V
18030306O	Murcia	Cucumber	>100 ^1^	>100 ^1^	R	R	A86V
18030306P	Murcia	Cucumber	>100 ^1^	>100 ^1^	R	R	A86V
18030306Q	Murcia	Cucumber	>100 ^1^	<100	R	MR	A86V
18030306S	Murcia	Cucumber	<50	>100 ^1^	LR	R	A86V
18030306U	Murcia	Cucumber	>100 ^1^	>100 ^1^	R	R	A86V
18020307A	Almeria	Cucumber	>100 ^1^	>100 ^1^	R	R	A86V
18020307B	Almeria	Cucumber	>100 ^1^	<100	R	MR	A86V
18020307C	Almeria	Cucumber	<50	<100	LR	MR	A86V
18020307D	Almeria	Cucumber	<50	<50	LR	LR	A86V
18020307E	Almeria	Cucumber	<50	>100 ^2^	LR	HR	A86V
18020307F	Almeria	Cucumber	<50	>100 ^1^	LR	R	A86V
18020307H	Almeria	Cucumber	<100	>100 ^1^	MR	R	A86V
18020307I	Almeria	Cucumber	>100 ^1^	0	R	S	G151R
18020307J	Almeria	Cucumber	<50	>100 ^2^	LR	HR	A86V
18020307K	Almeria	Cucumber	<50	>100 ^2^	LR	HR	A86V
18020307L	Almeria	Cucumber	<50	>100 ^1^	LR	R	A86V
18020307M	Almeria	Cucumber	<50	>100 ^2^	LR	HR	A86V
18020307N	Almeria	Cucumber	<50	<100	LR	MR	A86V
18020307P	Almeria	Cucumber	<50	>100 ^1^	LR	R	A86V
18020307Q	Almeria	Cucumber	<50	>100 ^1^	LR	R	A86V
18020307R	Almeria	Cucumber	<50	>100 ^1^	LR	R	A86V
18020307S	Almeria	Cucumber	<100	>100 ^1^	MR	R	A86V
18020307T	Almeria	Cucumber	<50	>100 ^1^	LR	R	A86V
18020208B	Murcia	Zucchini	<50	>100 ^2^	LR	HR	A86V
18020208D	Murcia	Zucchini	>100 ^1^	>100 ^1^	R	R	A86V
19020203A	Almeria	Zucchini	<100	>100 ^2^	MR	HR	A86V
19020203B	Almeria	Zucchini	<50	>100 ^1^	LR	R	A86V
19020203C	Almeria	Zucchini	<50	>100 ^1^	LR	R	A86V
19020203F	Almeria	Zucchini	>100 ^1^	>100 ^2^	R	HR	A86V
19020203H	Almeria	Zucchini	<100	>100 ^1^	MR	R	A86V
19020203I	Almeria	Zucchini	<50	>100 ^2^	LR	HR	A86V
19020203J	Almeria	Zucchini	<50	>100 ^1^	LR	R	A86V
19020304A	Almeria	Cucumber	<50	>100 ^1^	LR	R	A86V
19020304B	Almeria	Cucumber	0	>100 ^2^	S	HR	A86V
19020304C	Almeria	Cucumber	0	>100 ^2^	S	HR	A86V
19020304D	Almeria	Cucumber	0	>100 ^2^	S	HR	A86V
19020304G	Almeria	Cucumber	0	>100 ^1^	S	R	A86V
19020304I	Almeria	Cucumber	0	>100 ^1^	S	R	A86V
19020304J	Almeria	Cucumber	0	>100 ^1^	S	R	A86V
19020304O	Almeria	Cucumber	0	>100 ^1^	S	R	A86V
19020304S	Almeria	Cucumber	0	>100 ^1^	S	R	A86V

^1^ Normal growth of the mycelium. ^2^ Vigorous growth of the mycelium. Phenotypes are indicated as: S, sensitive; LR, low resistance; MR, moderate resistance; R, resistant; HR, high resistance.

**Table 6:** Tests and results of LAMP assay for detecting the amino acid changes A86V and G151R in *P. xanthii*.

Optimization of LAMP Reaction					Repeatability					Spores Assay			Field Samples Assay		
	t (min:s) ^1^	T (°C) ^1^	t (min:s) ^1^	T (°C) ^1^		Phenotype	Genotype	t (min:s) ^1^	T (°C) ^1^		t (min:s) ^1^	T (°C) ^1^		t (min:s) ^1^	T (°C) ^1^
Set 1-A86V															
	19020304D		SF9												
60.5 °C	27:30	87.44	-	-	18020303T	B^R^F^R^	A86V	16:30	85.50	19020304D	20:30	85.70	Leaf 1–3	29:15	85.99
61.6 °C	26:00	87.64	-	-	18020305L	B^MR^F^R^	A86V	14:00	85.79	SF9	-	-	Leaf 4–6	28:30	86.19
62.7 °C	23:00	87.10	-	-	18030306D	B^MR^F^MR^	A86V	14:30	85.70	g19020304D ^2^	15:30	85.50	Leaf 7–8	28:45	86.39
63.8 °C	22:30	86.98	-	-	18030306M	B^MR^F^R^	A86V	15:45	85.79	gSF9 ^2^	-	-	Leaf 9–10	27:00	85.99
64.9 °C	22:00	87.68	-	-	18020307K	B^LR^F^HR^	A86V	14:45	85.30				19020304D	23:30	86.19
66 °C	23:15	87.68	-	-	18020307L	B^LR^F^R^	A86V	15:15	85.79				g19020304D ^2^	15:45	85.40
67.1 °C	-	-	-	-	19020304D	B^LR^F^R^	A86V	14:45	85.20				gSF9 ^2^	-	-
69.5 °C	-	-	-	-	MR03	B^S^F^S^	-	-	-						
					SF60	B^S^F^S^	-	-	-						
					SF9	B^S^F^S^	-	-	-						
					311271	B^S^F^S^	-	-	-						
Set 4-G151R															
	18020307I		SF9												
60.5 °C	-	-	-	-	18030306K	B^R^F^LR^	G151R	22:00	85.10	18020307I	22:15	85.30	Leaf 1–3	-	-
61.6 °C	25:15	87.44	-	-	18020307I	B^R^F^S^	G151R	21:45	85.30	SF9	-	-	Leaf 4–6	-	-
62.7 °C	24:30	87.64	-	-	MR03	B^S^F^S^	-	-	-	g18020307I ^2^	20:45	85.90	Leaf 7–8	-	-
63.8 °C	25:15	87.10	-	-	SF60	B^S^F^S^	-	-	-	gSF9 ^2^	-	-	Leaf 9–10	-	-
64.9 °C	27:45	86.98	-	-	SF9	B^S^F^S^	-	-	-				18020307I	21:00	85.50
66 °C	28:00	87.68	-	-	311271	B^S^F^S^	-	-	-				g18020307I ^2^	20:30	85.70
67.1 °C	-	-	-	-									gSF9 ^2^	-	-
69.5 °C	-	-	-	-											

^1^ t refers to the amplification time in minutes:seconds and T to the melting temperature in Celsius, ^2^ Genomic DNA used extracted using conventional methods. For negative results, - is indicated.
